# Molecular Characterization of the Insulin-Like Androgenic Gland Hormone in the Swimming Crab, *Portunus trituberculatus*, and Its Involvement in the Insulin Signaling System

**DOI:** 10.3389/fendo.2020.00585

**Published:** 2020-09-02

**Authors:** Qinghua Jiang, Hongkun Zheng, Liang Zheng, Yaojing Wang, Mengen Wang, Xi Xie, Dongfa Zhu

**Affiliations:** Key Laboratory of Applied Marine Biotechnology of Ministry of Education, Ningbo University, Ningbo, China

**Keywords:** insulin-like androgenic gland hormone, cDNA clone, RNAi, eyestalk ablation, insulin signaling

## Abstract

The insulin-like androgenic gland hormone (IAG) is mainly produced in the androgenic gland (AG) of the male crustaceans and is a crucial regulator in male sexual differentiation. In the current study, the full-length cDNA of IAG in the swimming crab, *Portunus trituberculatus* (*Pt-IAG*), was cloned and characterized. Similar to other reported IAGs, the deduced amino acid sequence of Pt-IAG consists of signal peptide, B chain, C peptide, and A chain, containing six conserved cysteines that form two interchain disulfide bonds and one intra-B chain disulfide bond. Tissue distribution analysis suggested that the *Pt-IAG* cDNA was highly expressed in the AG and was slightly expressed in several other tissues. A short-term silencing of *PtIAG* with double-stranded RNA was found to reduce the transcript levels of insulin receptor (Pt-IR) and insulin-like growth factor-binding protein (Pt-IGFBP), suggesting the Pt-IAG might perform its biological function through the insulin family-based signaling system. Bilateral eyestalk ablation (ESA) induced the expression of *Pt-IAG* in the AG at 4 and 7 days after surgery, while the transcript levels of *Pt-IR* in the AG and testis and *Pt-IGFBP* in the muscle, testis, and thoracalia ganglia were significantly decreased from 1 day after surgery. The results suggested that the *Pt-IR* and *Pt-IGFBP* might also be the targets of eyestalk neuropeptides and responded to the ESA independent of IAG regulation.

## Introduction

The insulin-like androgenic gland hormone (IAG) is an insulin-like hormone mainly produced in the androgenic gland (AG) of male crustaceans. As a crucial regulator in crustacean male sexual differentiation, IAG has been widely identified in various crustacean species, and its function has been firmly established by numerous studies, such as microsurgical removal or implantation of AG, injection of hypertrophic AG cells, and IAG silencing [see review in (1, 2)]. Besides its role in sexual development, IAG was also proposed to participate in the process of growth (3), glucose metabolism (4), and ovarian development (5).

Compared to the molecular characterization and functional analysis, the mechanisms on how IAG works were much less studied. Some early studies had noticed that eyestalk ablation (ESA) can lead to the hypertrophy and hyperactivity of AG, and it was eventually proposed that IAG production may be negatively regulated by the inhibitory neurohormone in the sinus gland of eyestalk through an eyestalk–AG endocrine axis (6). The hypothesis was further supported by several studies showing an inhibitory role of specific eyestalk neuropeptides on IAG expression, such as gonad-inhibiting hormone (GIH) and molt-inhibiting hormone (MIH) in *Macrobrachium nipponense* (7) and crustacean female sex hormone (CFSH) in *Scylla paramamosain* (8).

Khalaila et al. (6) also found that the AG secretory products can directly activate protein kinases and phosphatases of some testicular polypeptides. This suggests that the signal transduction of IAG may work resembling the insulin family-based signaling system, which was well-described in vertebrates. It has been revealed in some pioneer studies that IAG can act as an active ligand to the insulin receptor (IR) (9, 10), which is responsible for transducing the insulin or insulin-like peptide (ILP) signals from the intercellular to the intracellular environment (11). In a recent work, long-term knockdown of IR in *Macrobrachium rosenbergii* by injection siRNA successfully yielded neo-females, suggesting the essential role of IR in IAG functions (12). IAG also showed a potential in interacting with the insulin-like growth factor-binding protein (IGFBP), as demonstrated in studies involving the binding assays (13), RNAi (14), and *in vitro* studies (15). IGFBPs act as the “carriers” and “reservoirs” of IGFs, modulating their availability and activity (16). All the IGFBPs identified in crustaceans are structurally similar to the IGFBP-related proteins (IGFBP-rPs, also named IGFBP7) (15), that binds insulin with higher affinities. This would be compatible with the fact that the structure of IAG is more similar to insulin than IGFs.

The swimming crab, *Portunus trituberculatus*, is a commercial species that has been extensively artificially propagated and cultivated in Chinese water. Monosex culture is considered an attractive approach for gaining higher yields of commercial crustaceans during both breeding and sailing processes. Despite this, the basic knowledge of IAG in this species is still lacking. The present study reported the first cloning of the IAG gene in *P. trituberculatus* (*Pt-IAG*), in parallel to the determination of its mRNA levels in different tissues and developmental stages. To verify the involvement of *Pt-IAG* in the insulin-signaling system, a dsRNA-mediated RNAi of *Pt-IAG* was utilized to evaluate its regulation on the expression of putative *Pt-IR* and *Pt-IGFBP* genes. Furthermore, as the eyestalk–AG endocrine axis exists, ESA surgery was performed to investigate the response of the *Pt-IAG, Pt-IR*, and *Pt-IGFBP*.

## Materials and Methods

The full-length cDNA of the *Pt-IAG* was cloned using RT-PCR and rapid amplification of 5′ complementary DNA ends (RACE). Briefly, a fragment of *Pt-IAG* was firstly obtained using a pair of degenerate primers (Table 1). Then, the 3′ and 5′-ends of *Pt-IAG* were obtained according to the manufacturer's protocol of SMARTer^TM^ RACE cDNA Amplification Kit (Clontech). The sequencing results from the above fragments were spliced using Vector NTI 10.0 software. The accuracy of the splicing sequence was confirmed by a long PCR using a pair of primers covering all the predicted open reading frame (ORF) regions. The nucleotide sequences of *Pt-IAG* and its deduced amino acid sequences were compared to the known decapod IAGs from NCBI database using the Clustal W multiple sequence alignment program. A phylogenetic tree was generated with the neighbor-joining option of molecular evolutionary genetics analysis (MEGA) version 5.0 by multiple sequence alignment with 16 known sequences from the NCBI database. Bootstrap analysis of 1,000 replicates was carried out to determine the confidence of tree branch positions.

**Table 1:** Primers used in this study.

**Name**	**Sequence (5′-3′)**	**Purpose**
*DP-F*	CCGACTTCTCCGTGGACTGYGGNAAYYT	RT-PCR
*DP-R*	GGGCCGCAGGGTGTCRCARTAYTC	RT-PCR
*Pt-IAG-3F1*	TTCGCAGATCCCACCGGAA	3′ RACE
*Pt-IAG-3F2*	AATGTTGCCCGCAGTCCAC	3′ RACE
*Pt-IAG-5R1*	CGGGCAACATTCGTCATA	5′ RACE
*Pt-IAG-5R2*	CTGCGAATCCTTCTTCCTATCC	5′ RACE
*Pt-IAG-VF*	GTCCTCACCAAGAATGTGCCTG	Long PCR
*Pt-IAG-VR*	CTTCCTCTTACTGCCTATTTCGGG	Long PCR
*Pt-IAG-qF*	TCTTATTAGCGACTTCTCCG	qPCR
*Pt-IAG-qR*	CCTCTGTCCCTCGTTTATGT	qPCR
*Pt-IR-qF*	AGAAGGTGCCCAGGAACTAAA	qPCR
*Pt-IR-qF*	AGGTGAGGTTGGATCGGAAT	qPCR
*Pt-IGFBP-qF*	TTACCACTATTGACGGCACCT	qPCR
*Pt-IGFBP-qR*	TCATTATC TGTACCCATCCTGTT	qPCR
*β-Actin-F*	CGAAACCTTCAACACTCCCG	qPCR
*β-Actin-R*	GATAGCGTGAGGAAGGGCATA	qPCR
*Pt-IAG-IF*	TAATACGACTCACTATAGGGTCTTATTAGCGACTTCTCCG	RNAi
*Pt-IAG-IR*	TAATACGACTCACTATAGGGCGTTGTCCTCATCCTCCT	RNAi
*GFP-IF*	TAATACGACTCACTATAGGGCGACGTAAACGGCCACAAGT	RNAi
*GFP-IR*	TAATACGACTCACTATAGGGCTTGTACAGCTCGTCCATGC	RNAi

*The T7 promoter sequence was underlined*.

For tissue distribution analysis, wild adult swimming crabs (four males with body weight of 164–209 g and carapace width of 13.4–15.1 cm and four females with body weight of 269–275 g and carapace width of 16.8–17.2 cm) were purchased from the local aquatic market in Zhenhai, Ningbo. Tissues including androgenic gland, testis, distal spermatic duct, ejaculatory bulb, gill, heart, eyestalk, Y-organ, mandibular organ, thoracic ganglion, hepatopancreas, ovary, muscle, brain, and intestine were dissected on ice and then stored in RNA preservation fluid (Cwbiotech) at −80°C.

For the RNAi experiment, the dsRNA was synthesized according to the instruction of MEGAscript T7 Kit (Ambion). Briefly, 446 bp of Pt-IAG and 655-bp green fluorescent protein (GFP) amplicons were cloned into pMD18-T vector (Takara) and amplified by PCR with a T7 promoter linked primer (Table 1). The resultant DNAs were used as the templates to synthesize dsRNA in a 20-μl *in vitro* transcription system. Integrity of the dsRNA was checked on agarose gel, and the concentrations of dsRNA were determined using a NanoDrop 2000 UV Spectrophotometer (Thermo Fisher). For the *in vivo* dsRNA injection, healthy juvenile male crabs (C5 juveniles, body weight 0.8–1 g) were divided into two groups, the *Pt-IAG* dsRNA injection group and the GFP dsRNA injection group. Then, 3 μg of dsRNA was injected via the base of the last walking leg with 10-μl syringe. Five crabs in each injection group were sacrificed at 0, 12, 24, 48, and 96 h post-injection, respectively, and then placed into the RNA preservation fluid (Cwbiotech) at −80°C until used.

For the ESA experiment, male swimming crabs (50–80 g) were temporarily reared for 7 days before ESA. The crabs were divided into the initial control group, the concurrent control (non-surgery) group, and the ESA group. Initial and concurrent controls received no treatments, while the initial controls (*n* = 4) were sacrificed on the first day of the experiment. ESA group received bilateral ablation of their eyestalks using sharp sterile scissors. To minimize hemolymph loss and infection, the wounds were cauterized with flame-heated sharp spatula. Four crabs in each of the ESA and concurrent groups were sacrificed at 0, 12, 24, 48, and 96 h post-injection. Tissues including AG, testis, muscle, and thoracalia ganglia were dissected on ice and stored in RNA preservation fluid (Cwbiotech) at −80°C until used.

Gene expression levels in this study were determined using quantitative real-time PCR (qPCR). qPCR primers for *Pt-IAG, Pt-IR, Pt-IGFBP*, and the reference gene β-actin were listed in Table 1. PCR was carried out using the SYBR®Premix Ex Taq^TM^ II kit (Takara) according to manufacturer's instructions. Relative mRNA expression levels normalized to β-actin were calculated by the comparative Ct (2^−ΔΔCt^) method (17). Transcript abundance in a representative replicate was set as the calibrator. All other quantities were expressed as an n-fold difference relative to the calibrator. The statistical significance in this study was analyzed using the SPSS 19.0 software. All data were subjected to the normality test using the Kolmogorov–Smirnov and Cochran tests prior to all statistical tests. Significant differences were accepted at *P* < 0.05 using one-way ANOVA followed by Student's *t*-test or Tukey test.

## Results

**Figure 1:**
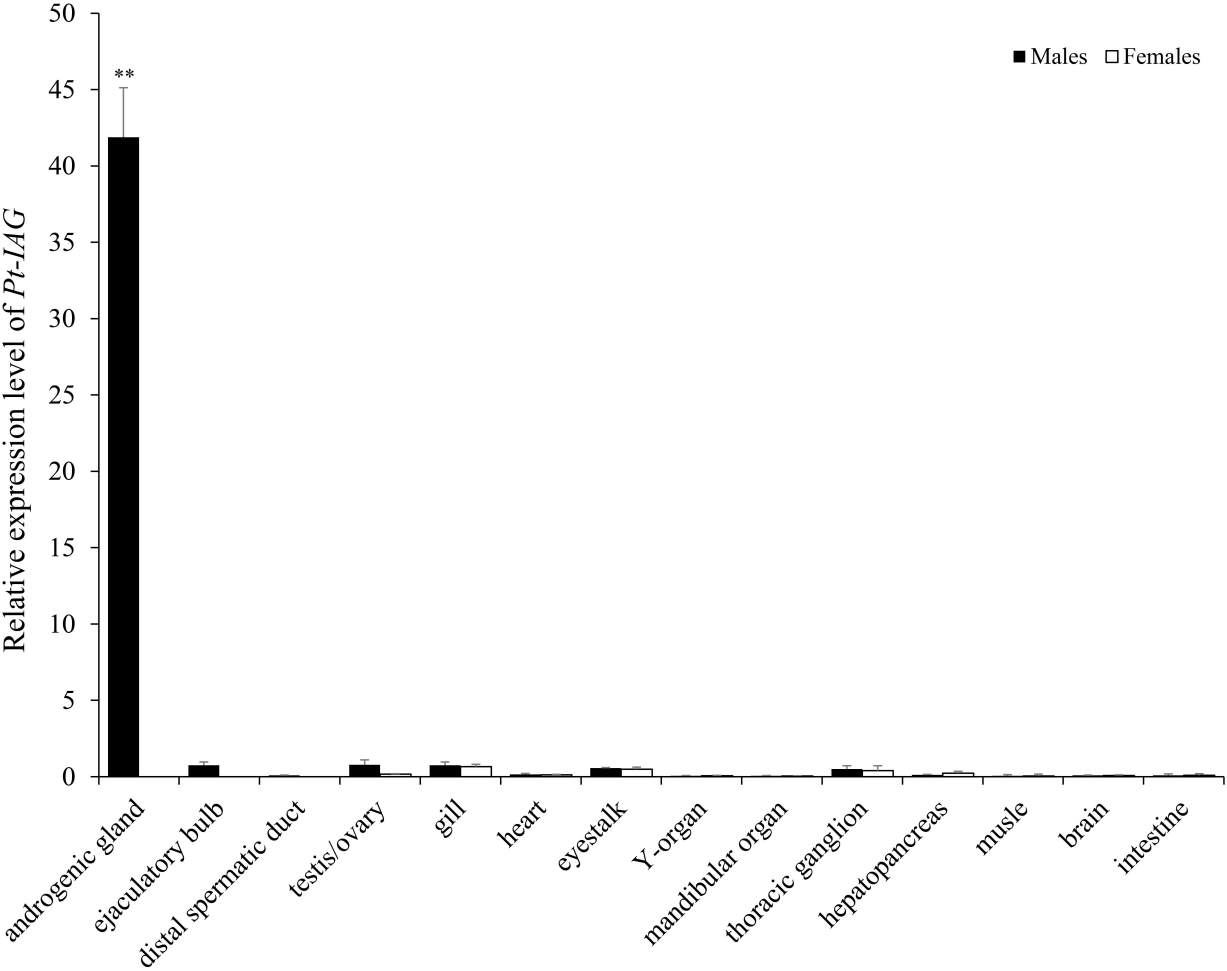
Tissue distribution of the insulin-like androgenic gland hormone of *Portunus trituberculatus* (*Pt-IAG*) transcripts. Bars represent mean ± SEM (*n* = 4). **Values statistically different from other groups (*P* < 0.01, Student's *t*-test).

The full-length cDNA of the *Pt-IAG* (GenBank accession number: KX168425) was 1,126 bp in length, which encoded 153 amino acid (aa), consisting of a signal peptide of 19 aa, a B chain of 32 aa, a C peptide of 57 aa, and an A chain of 45 aa. Like other known IAGs, the deduced aa sequence of Pt-IAG contained a putative N-glycosylation site (NCT), two putative cleavage sites (RHKR and RIRR), and six conserved cysteine residues. The Pt-IAG was clustered into decapods clade in the phylogenetic tree and was more closely related to Sp-IAG and Cs-IAG than to other IAGs. Tissue distribution analysis showed that the *Pt-IAG* mRNA was highly expressed in the AG and slightly expressed in the testis, ejaculatory bulb, gill, eyestalk, mandibular organ, thoracic ganglion, and hepatopancreas (Figure 1).

**Figure 2:**
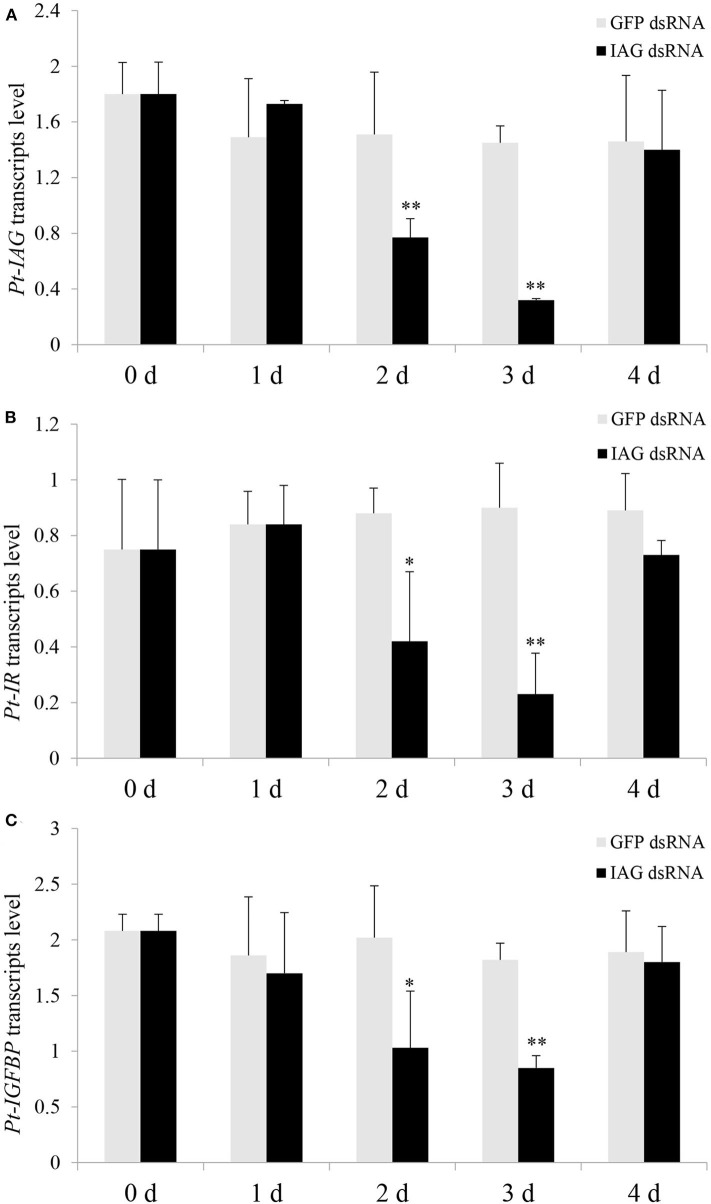
Effects of insulin-like androgenic gland hormone (IAG) dsRNA injection on gene expression of *Portunus trituberculatus* (*Pt*)*-IAG* **(A)**, *Pt-*insulin receptor (*IR*) **(B)**, and *Pt-*insulin-like growth factor-binding protein (*IGFBP*) **(C)**. Bars represent mean ± SEM (*n* = 5). **P < 0.01 or *P < 0.05 values statistically different from those of the control groups (Student's *t*-test).

Injection of the *Pt-IAG* dsRNA caused a significant decrease in *Pt-IAG* expression at 2 and 3 days post-treatment compared to that in the GFP dsRNA group, indicating good RNAi efficiency (Figure 2A). Injection of *Pt-IAG* dsRNA also led to a similar reduction in the mRNA levels of *Pt-IR* and *Pt-IGFBP* at 2 and 3 days post-injection (Figures 2B,C). The expression of *Pt-IAG, Pt-IR*, and *Pt-IGFBP* recovered to normal levels at 4 days post-injection, suggesting that the silencing effects are transitory.

**Figure 3:**
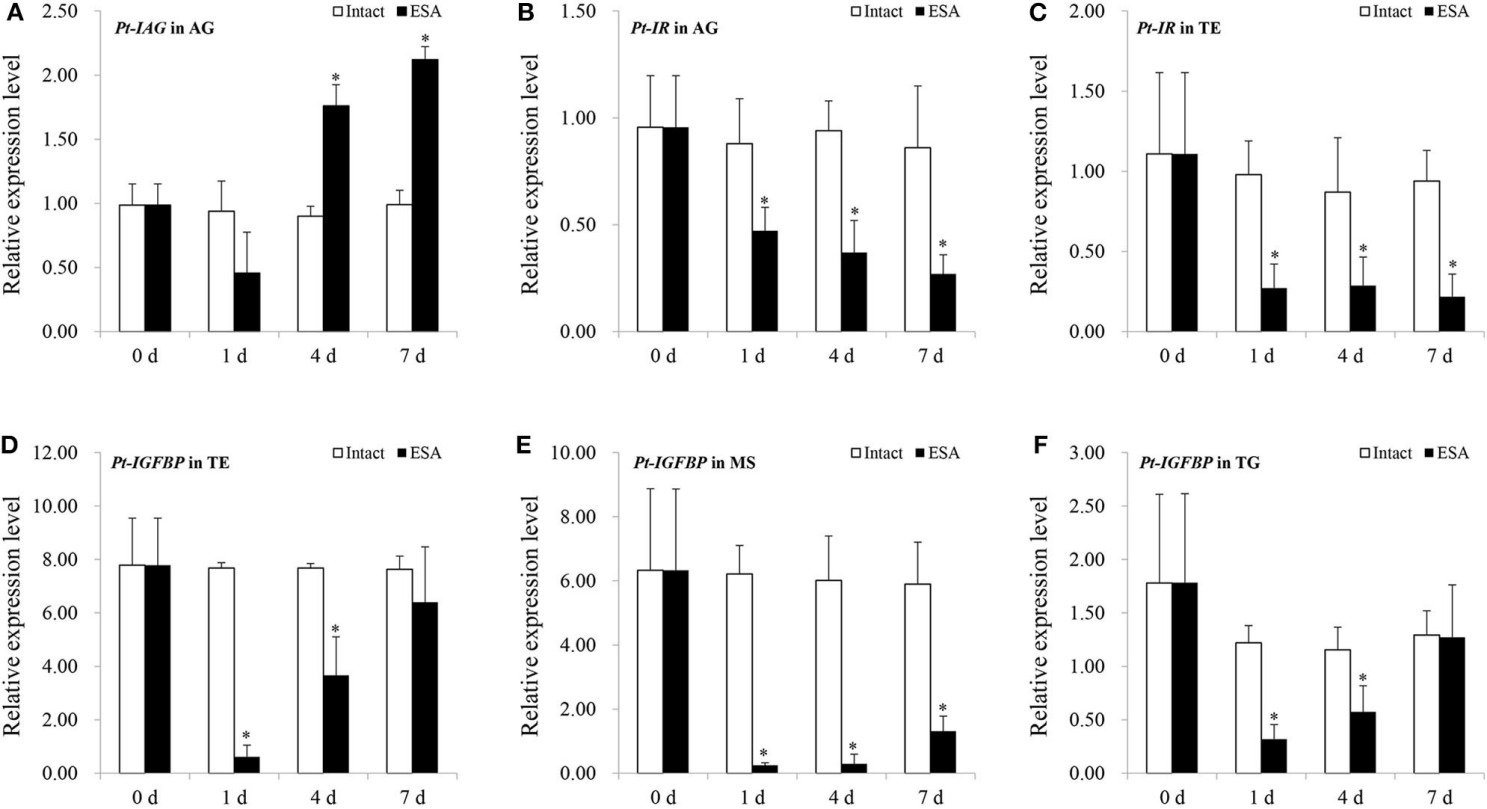
Effects of eyestalk ablation (ESA) surgery on the gene expression of insulin-like androgenic gland hormone of *Portunus trituberculatus* (*Pt-IAG*) in the androgenic gland (AG) **(A)**, *Pt-*insulin receptor (*IR*) in the AG **(B)**, and testis **(C)**, and *Pt-*insulin-like growth factor-binding protein (*IGFBP*) in the testis **(D)**, muscle **(E)**, and thoracalia ganglia **(F)**. Bars represent mean ± SEM (*n* = 4). **P < 0.01 represent values statistically different from those of the control groups (Student's *t*-test).

In the ESA experiment, the *Pt-IAG* expression in the AG was significantly induced from 4 to 7 days after the surgery (Figure 3A). By contrast, the expressions of *Pt-IR* and *Pt-IGFBP* were significantly decreased by ESA surgery. The *Pt-IR* expression in the AG and testis and the *Pt-IGFBP* expression in the testis, muscle, and thoracalia ganglia were examined. The test tissues were selected according to our preliminary data (unpublished), which were the main tissues of *Pt-IR* and *Pt-IGFBP* expression. For the *Pt-IR*, its mRNA level in the AG and testis decreased from 1 to 7 days after the surgery (Figures 3B,C). A similar pattern was also found for the *Pt-IGFBP* expression in the muscle. In the testis and thoracalia ganglia, the expression of *Pt-IGFBP* decreased from 1 to 4 days but recovered to normal level at 7 days after the surgery (Figures 3D–F).

## Discussion

The presented study reported the first study of the IAG gene in the swimming crab, *P. trituberculatus* (*Pt-IAG*). qPCR analysis showed a predominant expression of *Pt-IAG* in the AG, which is consistent with all the previous studies. Although the *Pt-IAG* transcripts were slightly expressed in the testis, ejaculatory bulb, gill, eyestalk, mandibular organ, thoracic ganglion, and hepatopancreas, it conforms to the recent consensus that the IAG is widely distributed among tissues, playing a broader role other than regulating sexual development. For instance, RNAi of IAG not only prevented the regeneration of male secondary sexual characteristics of *M. rosenbergii* but also led to a delay in molting and a reduction in growth (3). In the blue crab, *Callinectes sapidus, in vivo* silencing of hepatopancreas source IAG resulted in higher levels of hemolymph glucose than the control group, accompanied by significantly lower amounts of carbohydrate in the hepatopancreas, indicating a function of IAG in carbohydrate metabolism (4). Additionally, the IAG transcripts were also found in the ovary of *C. sapidus* and *S. paramamosain*, therefore being deduced as a regulator in ovarian development (5, 18).

To better understand the multiple functions of IAG, it is necessary to clarify the mechanisms of how IAG is transported to and is recognized by target tissues. An insulin family-based signaling system has been proposed based on the structure similarity of IAG to the insulin/IGF family. The hypothesis was further supported by several assays showing connections between IAG with the transmembrane IR (9, 10, 19) and the IGFBPs (13–15). The present study could provide another evidence for strengthening this hypothesis, concluded from the RNAi experiments showing a reduction in *Pt-IR* and *Pt-IGFBP* expression after injecting the *Pt-IAG* dsRNA. It is reasonable to speculate that the silencing in *Pt-IAG* might reduce the IAG signals, lessening the response of the insulin signaling system, thereby affecting the expression of *Pt-IR* and *Pt-IGFBP*.

ESA surgery has been extensively demonstrated to stimulate the IAG expression (7, 20–22), and this could also be verified by the ESA experiment in the present study. It was found that ESA also affected the expression of *Pt-IR* and *Pt-IGFBP*, which could be revealed by their decreased transcript levels in the tested tissues of surgery groups. The reduction in *Pt-IR* and *Pt-IGFBP* expression occurred earlier than the induction in *Pt-IAG* expression, suggesting that they responded to ESA independent of IAG regulation. In *M. rosenbergii*, IR silencing led to the hypertrophy of AG and the hyperactivity of Mr-IAG (10). In this respect, it is possible that the reduced expression of *Pt-IR* by ESA might in turn contribute to the subsequent increase in *Pt-IAG* expression.

In summary, the present study cloned the full-length cDNA of IAG in the swimming crab, *P. trituberculatus*. The Pt-IAG shared similarities in molecular characteristics and expression patterns to the IAGs that have been identified. *In vivo* silencing of the *Pt-IAG* reduced the transcript levels of *Pt-IR* and *Pt-IGFBP*, suggesting its involvement in the insulin family-based signaling system. Furthermore, the ESA experiments suggested that the *Pt-IR* and *Pt-IGFBP* might also be the targets of eyestalk neuropeptides and responded to the ESA independent of IAG regulation.

## Data Availability Statement

The datasets presented in this study can be found in online repositories. The names of the repository/repositories and accession number(s) can be found in the article.

## Author Contributions

DZ designed the study and wrote the manuscript. QJ contributed to the experimental work and integrated the data. HZ prepared the dsRNA and participated in tissue collection. LZ did the ESA surgery and participated in tissue collection. YW did the qPCR and participated in tissue collection. MW did the qPCR and ESA surgery. XX did the dsRNA injection and participated in tissue collection. All authors contributed to the article and approved the submitted version.

## Conflict of Interest

The authors declare that the research was conducted in the absence of any commercial or financial relationships that could be construed as a potential conflict of interest.
